# Accidental hypothermia: direct evidence for consciousness as a marker of cardiac arrest risk in the acute assessment of cold patients

**DOI:** 10.1186/s13049-022-01000-w

**Published:** 2022-03-04

**Authors:** Samuel Barrow, Galen Ives

**Affiliations:** 1 Royal Army Medical Corps, British Army, DMS Whittington, Lichfield, WS14 9PY UK; 2 Information School, University of Sheffield, Regent Court, 211 Portobello Street, Sheffield, S1 4DP UK

## Abstract

**Background:**

Rapid stratification of the risk of cardiac arrest is essential in the assessment of patients with isolated accidental hypothermia. Traditional methods based on measurement of core temperature are unreliable in the field. Behavioural observations have been used as predictors of core temperature and thus indirect predictors of cardiac arrest. This study aims to quantify the direct relationship between observed conscious level and cardiac arrest.

**Methods:**

Retrospective case report analysis identified 114 cases of isolated accidental hypothermia meeting inclusion criteria. Level of consciousness in the acute assessment and management phase was classified using the AVPU system with an additional category of “Alert with confusion”; statistical analysis then related level of consciousness to incidence of cardiac arrest.

**Results:**

All patients who subsequently suffered cardiac arrest showed some impairment of consciousness (*p* <  < .0001), and the risk of arrest increased directly with the level of impairment; none of the 33 fully alert patients arrested. In the lowest impairment category, *Alert confused*, a quarter of the 12 patients went on to arrest, while in the highest *Unresponsive* category, two thirds of the 43 patients arrested. Where core temperature was available (62 cases), prediction of arrest by consciousness level was at least as good as prediction from core temperature.

**Conclusions:**

This study provides retrospective analytical evidence that consciousness level is a valid predictor of cardiac arrest risk in isolated accidental hypothermia; the importance of including confusion as a criterion is a new finding. This study suggests the use of consciousness alone may be at least as good as core temperature in cardiac arrest risk prediction. These results are likely to be of particular relevance to the management of accidental hypothermia in the pre-hospital and mass casualty environment, allowing for rapid and accurate triage of hypothermic patients.

## Introduction

It is well established that patients with isolated accidental hypothermia are at risk of cardiac arrest [1]. In England and Wales between 2013 and 2018 there were 330 deaths from ‘exposure to excessive natural cold’ (Office for National Statistics), whilst in the United States an average of 1301 deaths/year occurred between 1999 and 2011 [2]. The fundamental cause of death in hypothermia is the level of critical brain hypoxia. The fall in brain oxygen-consumption at lower core temperatures provides some resilience to low or no cardiac output states [1, 3, 4], but the brain cannot withstand hypothermic cardiac arrest indefinitely. It is therefore vital to try to maintain any ongoing cardiac output during initial patient management.

Hypothermic patients have a susceptibility to ‘rescue-collapse’. Traditionally described in water rescue, this sudden deterioration of the patient on initial management by first responders is frequently seen in the land environment [5–7]. The pathophysiology behind rescue-collapse is not entirely understood, but is likely a combination of ‘afterdrop’, catecholamine changes, and movement induced arrhythmias in an unstable myocardium [8–16]. Rapid stratification of risk of cardiac arrest in these patients would have two main benefits. Firstly, accidental hypothermia is often encountered in remote areas including backcountry environments, military operations/exercises, and expeditions; the ability to efficiently triage hypothermic patients and decide who can be managed in the field with low risk of cardiac arrest is invaluable in appropriate logistic asset allocation. Secondly, having an early appreciation of cardiac arrest risk can guide initial management decisions towards maintaining a perfusing rhythm.

Traditionally, methods to stratify the risk of cardiac arrest have been based on core temperature [1, 17–19]. In the pre-hospital environment, reliable measurements of core temperature are often not accessible. Infrared tympanic probes are generally considered unsuitable for field use because the sensors are not calibrated in low core temperature [20]. Low reading core temperature thermometers may not be in use and, when available, many have methodological issues. Rectal probes require exposure and handling of the patient possibly resulting in further drops in core temperature (thermoneutral temperature for an unclothed human is 28 °C) [21] and/or movement induced arrhythmias [15]. In addition, rectal temperatures can lag behind true core temperature by up to an hour during warming and cooling and it has been suggested they are unsuitable for monitoring afterdrop [20, 22, 23]. Epitympanic thermistor probes may give falsely low values in patients with unstable circulation and require both modification and validation for pre-hospital use [4, 20, 24–26]. Oesophageal probe temperature, when placed in the lower 1/3 of the oesophagus, closely correlates with pulmonary artery temperature and should be the gold standard in patients with a secured airway [9]. However this is usually only appropriate for patients with reductions in consciousness. These factors, combined with the requirement to make rapid early management decisions, support the necessity for a clinical assessment tool as an adjunct to the initial patient evaluation.

Given the increased likelihood of cardiac arrest with lower core temperature [21], the well-known Swiss-Staging Model of hypothermia uses vital signs at presentation to estimate core temperature (as a surrogate for cardiac arrest risk) from clinical indicators only (Table 1) [19].

**Table 1:** Swiss staging of hypothermia, Brown et al. 2012 [27]

Stage	Brown et al. [27] symptoms/signs	Typical core temperature (°C)
HT I	Conscious, shivering	32 to 35
HT II	Impaired consciousness, not shivering	28 to < 32
HT III	Unconsciousness	24 to < 28
HT IV	No vital signs	< 24

There are a number of issues with this assessment tool. Its ability to estimate core temperature has been shown to be unsatisfactory [18]. Shivering is a notoriously unreliable sign, continuing with a core temperature at least down to 28–30 °C [23, 28]. Many studies have described patients with a core temperature of below 24 °C with vital signs [7, 29–31]. For these reasons the Wilderness Medical Society guidelines on accidental hypothermia, 2014 & 2019, advise against relying on the Swiss-Staging system [21, 32]. This is further supported by the most recent adaption of the Swiss System by the International Commission for Mountain Emergency Medicine (ICAR MedCom) [33].

Hypothermia is a clinical continuum with much interpersonal variation. Stratifying the risk of cardiac arrest by estimating the patient’s core temperature from the presenting clinical picture is not plausible because of the variance in individual response to specific core temperatures. Instead, directly predicting cardiac arrest risk from clinical features is preferable.

A retrospective case report analysis from Pasquier et al., reviewing 183 cases of accidental hypothermia for concordance between traditional Swiss-Staging classification and measured core temperature, demonstrated that no patients in their subgroup analysis with a normal conscious level (defined as ‘HT1’) suffered cardiac arrest [18]. However, given that their primary endpoint was the correspondence between core temperature and Swiss-Stage, no further conclusions regarding the use of conscious level as the predictor were drawn. In addition, Frei et al. undertook a systematic literature review of witnessed cardiac arrest in hypothermia to assess for clinical characteristics and outcomes in this patient group [34]. Crucially, all of the patients who had their GCS recorded (n = 24) who arrested had a GCS < 11—this GCS was referred to as ‘Pre-Cardiac Arrest GCS’ with no further information on timing of this assessment given.

The initial hypotheses of this study are therefore:

1. The risk of cardiac arrest is directly proportional to conscious level. Categorising hypothermia by level of consciousness is more appropriate and clinically repeatable for the management of hypothermic patients than traditional assessment by clinical estimation of core temperature.
2. The following proposed novel clinical stages will more accurately predict the risk of cardiac arrest, making management decisions easier, clearer and safer than previous assessment tools:
3. *Cold stressed with no risk of cardiac arrest* Patient Alert with normal cognition
4. *Hypothermia with low risk of cardiac arrest* Patient alert but has signs of altered cognition
5. *Hypothermia with significant risk of cardiac arrest* Patient has significant reductions in conscious level (V, P, U)

These hypotheses are partially supported by the recent ICAR MedCom ‘Revised Swiss-System’ [33]. This system applies a three point ordinal scale of ‘low’, ‘medium’, and ‘high’ risk of cardiac arrest based on conscious level as the primary element for staging, without attempt to quantify this risk further. The indirect argument presented is based on Pasquier et al’s study which demonstrates that haemodynamic and conscious level parameters correlate with core temperature, the latter showing the highest correlation (Spearman’s rho = 0.78) [35]. It is worth noting that this correlation is not the strongest, with a GCS of 5 being associated with a core temperature between 20 and 31 °C and a GCS as high as 14 being associated with a core temperature from 25 to 34 °C.

Testing the above hypotheses of this study aims to provide direct evidence for conscious level as an independent risk stratification tool in the initial assessment of patients with isolated accidental hypothermia. A retrospective case report analysis was conducted to look for correspondence between conscious level on arrival of medical support and cardiac arrest in the isolated accidental hypothermia patient group.

## Methods

A systematic literature review was undertaken to identify patients with isolated accidental hypothermia. No limit on publication date or status were set, but only studies in English were included. PubMed search keywords were limited to ‘‘case report’ AND ‘hypothermia’’. Studies were initially screened by title and abstract and if not excluded, the full paper was sourced and reviewed. A limited number of additional studies were sourced from the bibliography of included articles. PubMed ID was utilised to ensure studies were not duplicated. Patients were subsequently included/excluded based on specific criteria. Samples were included if patients were suitably suspected to have hypothermia on arrival of first responders. Samples were then excluded if the patient had already arrested on arrival of first responders; if the case was of iatrogenic/therapeutic/experimental hypothermia; if any additional factor was involved that may influence conscious level including alcohol, drugs, medical conditions, drowning (excluded due to influence of possible hypoxia on conscious level at assessment), trauma, learning disabilities, age < 4yo, avalanche without air pocket, medications outside therapeutic range, dementia; and if insufficient information was contained within the study.

### Data collection

The following variables were collected from included studies when available: author, date of publication, PubMed ID, patient age, sex, confounders, AVPU or comments on conscious level taken during patient assessment, timing of consciousness assessment where available, whether AVPU was documented or interpreted by researcher at time of reading, whether the patient was confused, initial GCS, initial systolic and diastolic blood pressure, initial heart rate, presence of arrhythmias, respiratory rate, initial core temperature, method of core temperature measurement, initial blood glucose, initial patient management, whether the patient suffered cardiac arrest, how cardiac arrest was confirmed, and any additional pertinent information.

Cases were classified into ‘Alert’, ‘Alert with confusion’, ‘Voice’, ‘Pain’ or ‘Unresponsive’ as follows: many studies (n = 45) directly stated the patient’s conscious-level which was accepted as published. A further set (n = 14) used the Glasgow Coma Scale which was converted into AVPU using criteria adapted from Kelly et al. and McNarry et al. [36, 37]. The remainder (n = 55) gave behavioural descriptions of the patient which required classifying. To avoid bias, this was conducted by the second author who was blinded to outcome (cardiac arrest or not) and any patient identifiable information including core temperature, age, or haemodynamic parameters. Patients were classified as follows:

- 'Alert’—Awake with normal cognitive function.
- ‘Alert Confused’—Alert but with a change in higher cognitive function such as unusual behaviour, inappropriate language, or inability to follow commands for non-physical reasons.
- ‘Voice’—Unalert responding with physical or verbal responses to verbal stimuli only.
- ‘Pain’—Unalert responding to physical stimuli only.
- ‘Unresponsive’—Responding to neither verbal nor painful stimuli

Core temperature data, where available, were classed as reliable if measured rectally, in the oesophagus, and in four cases, in the bladder. Timing of core temperature assessment was not always reported but was assumed to be during the acute assessment phase, including arrival to the emergency department, for all cases.

## Results

**Fig. 1:**
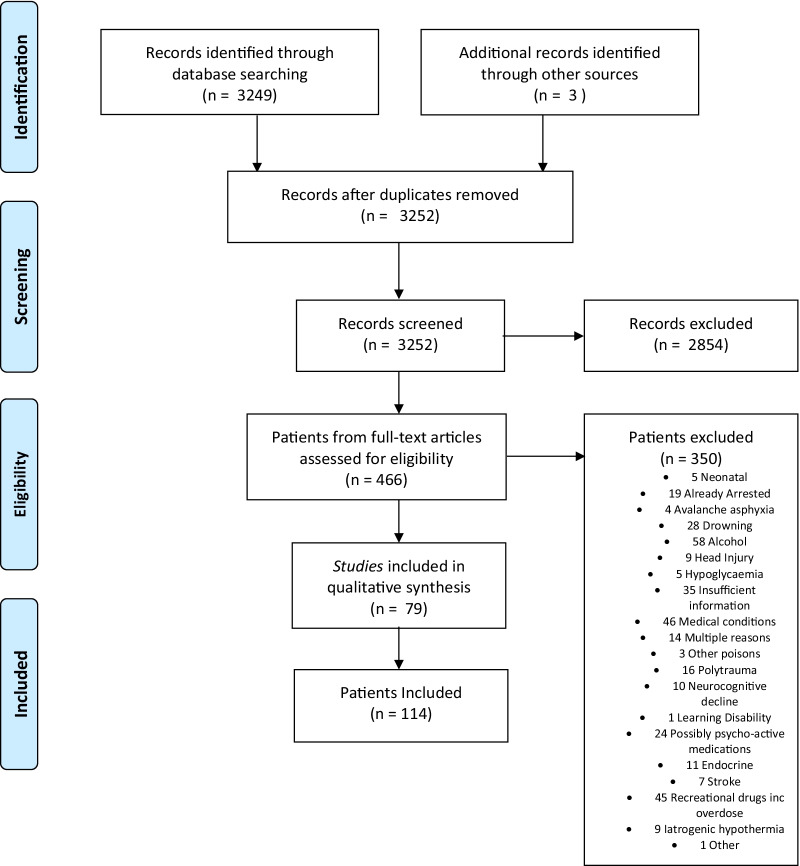
Data selection flow diagram

Of the 3252 records identified, 2854 were excluded by the title and abstract. 466 patient data sets were extracted from the remaining 398 full-text articles. These were in turn examined against inclusion and exclusion criteria resulting in 114 patient inclusions from 79 studies (Fig. 1).

The 114 cases examined comprised 37 females, 74 males and three for whom no gender was stated. Age was given for 104 cases (91%), with a mean of 43.0 years, range 4 to 95 years. Haemodynamic data were patchily presented, with all three parameters (SBP, DBP, HR) given in only 18 cases (16%). Presence or absence of arrhythmia was noted in 26 cases (23%). Reliable core temperature data were available for 61 cases (54%) and this subgroup was the subject of an additional analysis. Consciousness was assessed on initial arrival of first responders in 69 cases, on arrival to the Emergency Department in 41 cases, admission to Intensive Care in 1 case, and was unclear in 3 cases.

Of the 114 cases, 44 (39%) subsequently suffered a cardiac arrest. Table 2 summarises the observed differences between those who did, and did not, arrest.

**Table 2:** Comparison of *Arrest* and *No arrest* groups

	Cases	Sex	Mean age	Systolic BP	Diastolic BP	Heart rate	Arrhythmia present	Core temperature
Arrest	44	35% F 65% M	46.4	81.1	46.4	41.6	44%	23.5 °C
No arrest	70	30% F 70% M	40.6	98.4	66.0	56.7	25%	30.6 °C
N	114	111	104	30	20	42	26	84^†^
Significance		χ^2^ = 0.58 *p* = 0.68	t* = 1.16 *p* = .25	t* = 1.82 *p* = 0.08	t* = 3.07 *p* = 0.007	t* = 2.24 *p* = 0.03	Fisher Exact *p* = 0.42	t = 7.57 *p* < < 0.001

*Indicates t is corrected for unequal variance

^†^Includes the less reliable temperature measures

The two groups did not differ significantly by age or sex. Haemodynamic parameters differed in the expected direction and despite small numbers two of these, diastolic BP and heart rate, reached significance; where recorded, arrhythmia was not a discriminating factor in this sample. There was a large and highly significant difference in core temperature, as would be expected.

### Consciousness level

**Fig. 2:**
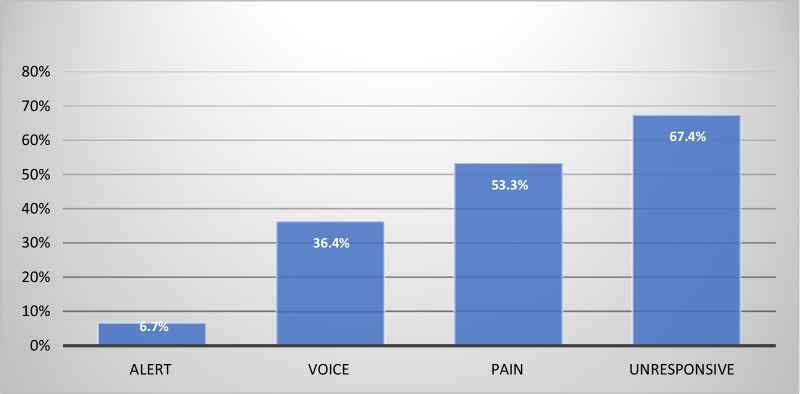
Percentage of cardiac arrests by consciousness level

There were large, systematic and highly significant differences in the reported level of consciousness between those who suffered cardiac arrest and those who did not, summarised in Table 3 and Fig. 2:

**Table 3:** Numbers of cardiac arrests by level of consciousness

	Arrest	No arrest	Totals
Alert	3	42	45
Voice	4	7	11
Pain	8	7	15
Unresponsive	29	14	43
Totals	44	70	114

The above differences are highly significant (χ^2^ = 35.9, *p* < 10^−7^)

### Confusion

Twelve of the 45 “Alert” cases were reported to show confusion, and all three of the “Alert” arrests occurred in this subgroup, shown in Table 4.

**Table 4:** Presence of confusion and occurrence of cardiac arrest

	Arrest	No arrest	Totals
No Confusion	0	33	33
Confused	3	9	12
Totals	3	42	45

The differences are significant, Fisher exact test *p* = 0.016

**Fig. 3:**
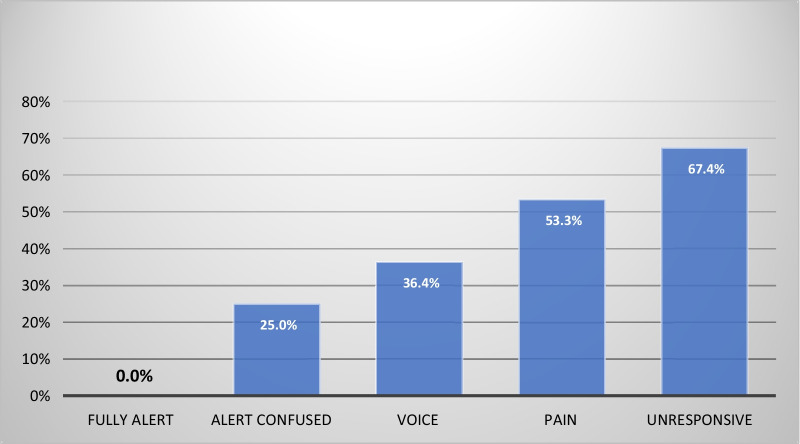
Percentage of cardiac arrests by consciousness level including confusion

Adding confusion as a discriminating factor alters Fig. 3 as shown below:

### Core temperature versus consciousness level as a discriminator

The subset of cases (62) for which reliable core temperature data were available allows comparison to be made between this criterion and consciousness level as a predictor of cardiac arrest (Tables 5, 6). The recommended core temperature threshold for predicting cardiac arrest is 28 °C [1, 21]. No alert cases were reported to be confused in this group, and the comparison below is between fully alert cases and the group comprising unresponsive cases and those responsive to pain/voice.

Whilst the two tables above are not statistically significant, the implication is that consciousness level predicts cardiac arrest at least as well, and potentially better, than core temperature, although possibly at the expense of marginally more false positives.

**Table 5:** Predictions from core temperature

	Predicted		
	Tem *p* < 28 °C	Temp > = 28 °C	Accuracy (%)
*Actual*			
Arrest	23	2	92
No arrest	13	24	65

**Table 6:** Predictions from consciousness level

	Predicted		
	V/P/U	A	Accuracy (%)
*Actual*			
Arrest	25	0	100
No arrest	18	19	51

## Discussion

The data set presented, which did not differ significantly by age or biological gender, showed highly significant differences in core temperature and conscious level between groups that did and did not suffer cardiac arrest. Haemodynamic data on assessment was sporadically reported, and only diastolic blood pressure and heart rate were significant. Systolic blood pressure and presence of arrhythmia were not significantly different. Caution should be taken in the interpretation of this haemodynamic data as absolute numbers were low and it may be expected that significantly unwell patients, who may tend more towards cardiac arrest, would have observations that are harder to gain and therefore be under-represented.

Grouping patients into ‘Alert’, ‘Voice’, ‘Pain’, and ‘Unresponsive’ (as described in the methods section), proved highly significant for cardiac arrest prediction. The subdivision of ‘Alert’ into ‘Alert’ and ‘Alert Confused’ was again highly significant. No patients in the ‘Alert’ and non-confused group arrested, whilst 25% of the ‘Alert’ but confused patients did arrest. These findings support the initial hypotheses of this study. It should be noted that *any* change in cognition proved to carry a significant risk of cardiac arrest (25%) and the more severe the change in consciousness the higher the risk of arrest.

Given that traditional risk assessment is based on core temperature, a sample with ‘reliable’ (defined above) core temperature measurement was extracted for analysis. The timing of core temperature measurement was not always available and was assumed to be during the acute phase of management in all cases (including initial assessment in ED). Core temperature proved a good predictor of arrest. Interestingly, Tables 5 and 6, whilst not statistically significant, suggest that conscious level may predict cardiac arrest risk at least as well as core temperature alone. However, due to robust population selection for core temperature acquisition, this population is fundamentally different from the main analysis population. In addition, the timing of core temperature measurement may not have corresponded with the timing of assessment of conscious level as this temporal relationship was rarely specifically stated in our population. Perhaps the combination of core temperature and consciousness, in the appropriate scenarios, could provide even more accurate prediction of cardiac arrest risk. Unfortunately, the sample size was insufficient to draw further conclusions.

The timing of the assessment of consciousness was on initial arrival of first responders in most cases (n = 69), with 41 cases being on arrival to the Emergency Department, 1 on arrival to Intensive Care, and was unclear in 3 cases. Therefore, practically, it seems pertinent to treat all patients with any change in cognition during the acute management (up to and including in the Emergency Department), where accidental hypothermia is suspected, with the utmost care in order to minimise cardiac arrest risk. For accidental hypothermia, conscious level may provide a practical monitoring tool in the pre-hospital cold weather space where traditional monitoring systems may be impractical or unreliable.

Taking into account the limitations discussed below, this provides direct evidence to support a move away from the traditional Swiss-Staging System towards utilising level of consciousness as a triage tool for assessing patients with isolated accidental hypothermia further to the ICAR MedCom ‘Revised Swiss System’ [33].

It would be interesting to consider the underlying physiological association between conscious level and susceptibility to cardiac arrest in accidental hypothermia. Whilst the cardiac and neurological changes associated with hypothermia are complex and multifactorial [38], perhaps an underlying metabolic/electrical susceptibility to cold may explain the vast variation in conscious level and cardiac arrest risk over various core temperatures.

## Limitations

As this study is a retrospective case report analysis, the risk of publication bias is present. However, due to the nature of accidental hypothermia, case reports were selected as the inclusion data type due to the paucity of experimental data on isolated significant hypothermia, and the desire to extrapolate this study to real world assessment cases. This study was also subject to language bias (only studies in English were included), and some degree of availability bias (limited to PubMed database search). The authors attempted to limit the impact of outcome bias by including all available case reports for analysis.

Accidental hypothermia is uncommon. Whilst this may tend somewhat against outcome reporting bias—given that cases are more likely to be published regardless of outcome due to the infrequency of disease occurrence—the statistical power of this study may be reduced due to small sample size.

Another important consideration is that the population analysed is highly selected to remove any confounders of conscious level. Some case reports may have excluded details of these confounders and may thus be mis-represented in the analysis.

Furthermore, as many cases of accidental hypothermia encountered in real world medical practice are multifactorial, extrapolation is to a distinct but relatively narrow field of application.

Another limitation is the timeline information in the data sample. Timings of assessment of consciousness, measurement of core temperature, and confirmation of cardiac arrest were rarely all published within a single case report. We therefore limit our interpretation to state that if conscious level decreases at any point in patient assessment or management, up to and including the Emergency Department, the risk of cardiac arrest increases. Furthermore, this dataset limits interpretation of the imminence of cardiac arrest after assessment. As only one study progressed to give information in the Intensive Care Unit, it can be assumed that the risk of arrest is present in the acute course.

In terms of data interpretation, the authors attempted to limit bias in the extraction of ‘AVPU’ categorisation from the 114 cases by blinding the second author to outcomes and patient information so ‘AVPU’ could be independently assessed. However three issues remain: Firstly, the 45 studies that directly state the patient’s conscious level are subject to the inherent interrater reliability issues with the AVPU scale [39]; Secondly, the 14 GCS conversions undertaken in this analysis are influenced by overlap in the categorisation of ‘V’ and ‘P’ by GCS (‘A’ and ‘U’ are more readily associated with GCS 15 and 3 respectively) [36, 37]; Thirdly, the 55 cases which required interpretation of ‘AVPU’ from descriptive text may be less reproducible and clinically accurate than cases with documented consciousness.

Regarding clinical data, there was generalised underreporting of the method of cardiac arrest confirmation. This is relevant because detecting cardiac output in the severely hypothermic, non-responsive patient can be extremely difficult, and further complicated by recent suggestions to withhold CPR in a possibly perfusing rhythm even in the absence of pulses (PEA) [21]. We may infer that whilst a number of studies explicitly stated the method of arrest confirmation (n = 27), some patients in which this was not stated may have been initially misdiagnosed as cardiac arrest.

Perhaps most importantly, there was wide variation in descriptions of the initial management of patients with suspected accidental hypothermia. The majority of studies provided insufficient information to control for appropriate initial management. It is likely that circum-rescue collapse and rescue induced cardiac arrest can be somewhat mitigated with careful handling, selective rewarming etc. [21]. Any patient management, therefore, conducted contrary to current hypothermia guidelines would carry a disproportionately high risk of arrest, skewing our results/interpretation.

It must be noted that this study is not a replacement for clinical judgement. Whilst it does provide retrospective evidence for the use of consciousness as a predictor of cardiac arrest in isolated accidental hypothermia, clinicians should use caution and draw on multiple sources in their assessment of the critically ill patient.

## Conclusion

This study provides retrospective analytical evidence that conscious level is a valid predictor of cardiac arrest risk in isolated accidental hypothermia. Any disturbance of consciousness during acute assessment and early management indicates a significant risk of cardiac arrest. As the patient becomes less conscious, the risk of cardiac arrest increases. Our data suggest that conscious level may be at least as good as core temperature in cardiac arrest risk stratification. This provides the possibility for a rapid simple risk assessment tool for use in the pre-hospital environment.

A number of studies have now pointed towards consciousness being an important predictor of arrest [18, 33, 34], and this study has specifically assessed this inference. Whilst there are limitations, this study provides the basis for future prospective study of ‘Alert’, ‘Alert-confused’, ‘Voice’, ‘Pain’, and ‘Unresponsive’ in the assessment of accidental hypothermia cardiac arrest risk.

## References

[1] Lott C, et al. European resuscitation council guidelines 2021: cardiac arrest in special circumstances. Resuscitation. 2021;161:152–219. 10.1016/j.resuscitation.2021.02.011. 33773826 10.1016/j.resuscitation.2021.02.011

[2] Xu J. QuickStats: number of hypothermia-related deaths,* by sex—National Vital Statistics System, United States,† 1999–2011§. Morbidity and mortality weekly report (MMWR); 2013.

[3] McCullough JN, et al. Cerebral metabolic suppression during hypothermic circulatory arrest in humans. Ann Thorac Surg. 1999. 10.1016/S0003-4975(99)00441-5. 10391334 10.1016/s0003-4975(99)00441-5

[4] Paal P, et al. Accidental hypothermia-an update. Scand J Trauma Resusc Emerg Med. 2016;24:1–20. 27633781 10.1186/s13049-016-0303-7 PMC5025630

[5] Althaus U, Aeberhard P, Schupbach P, Nachbur BH, Mühlemann W. Management of profound accidental hypothermia with cardiorespiratory arrest. Ann Surg. 1982. 10.1097/00000658-198204000-00018. 7065752 10.1097/00000658-198204000-00018 PMC1352533

[6] Golden FS, Hervey GR, Tipton MJ. Circum-rescue collapse: collapse, sometimes fatal, associated with rescue of immersion victims. J Roy Naval Med Serv. 1991;77:3. 1815081

[7] Oberhammer R, et al. Full recovery of an avalanche victim with profound hypothermia and prolonged cardiac arrest treated by extracorporeal re-warming. Resuscitation. 2008. 10.1016/j.resuscitation.2007.09.004. 17988783 10.1016/j.resuscitation.2007.09.004

[8] McIntosh SE, et al. Wilderness medical society practice guidelines for the prevention and treatment of frostbite: 2019 update. Wilderness Environ Med. 2019;30:19–32. 10.1016/j.wem.2019.05.002 31326282

[9] Hayward JS, Eckerson JD, Kemna D. Thermal and cardiovascular changes during three methods of resuscitation from mild hypothermia. Resuscitation. 1984. 10.1016/0300-9572(84)90031-5. 6322264 10.1016/0300-9572(84)90031-5

[10] Romet TT. Mechanism of afterdrop after cold water immersion. J Appl Physiol. 1988. 10.1152/jappl.1988.65.4.1535. 3182516 10.1152/jappl.1988.65.4.1535

[11] Giesbrecht GG, Bristow GK. A second postcooling afterdrop: more evidence for a convective mechanism. J Appl Physiol. 1992. 10.1152/jappl.1992.73.4.1253. 1447067 10.1152/jappl.1992.73.4.1253

[12] Stoneham MD, Squires SJ. Prolonged resuscitation in acute deep hypothermia. Anaesthesia. 1992. 10.1111/j.1365-2044.1992.tb03257.x. 1306059 10.1111/j.1365-2044.1992.tb03257.x

[13] Danzl DF. Chapter 5—Accidental Hypothermia. Wilderness Medicine, 6/e (2012). 10.1016/B978-1-4377-1678-8.00005-2.

[14] Lee CH, Van Gelder C, Burns K, Cone DC. Advanced cardiac life support and defibrillation in severe hypothermic cardiac arrest. Prehosp Emerg Care. 2009. 10.1080/10903120802471907. 19145531 10.1080/10903120802471907

[15] Giesbrecht GG, Hayward JS. Problems and complications with cold-water rescue. Wilderness Environ Med. 2006. 10.1580/PR01-05.1. 16538942 10.1580/pr01-05.1

[16] Vanggaard L, Eyolfson D, Xu X, Weseen G, Giesbrecht GG. Immersion of distal arms and legs in warm water (AVA rewarming) effectively rewarms mildly hypothermic humans. Aviat Sp Environ Med. 1999;70:1081–8. 10608605

[17] Vanden Hoek TL, et al. Part 12: cardiac arrest in special situations: 2010 American Heart Association Guidelines for Cardiopulmonary Resuscitation and Emergency Cardiovascular Care. Circulation. 2010. 10.1161/CIRCULATIONAHA.110.971069. 20956228 10.1161/CIRCULATIONAHA.110.971069

[18] Pasquier M, et al. An evaluation of the Swiss staging model for hypothermia using hospital cases and case reports from the literature. Scand J Trauma Resusc Emerg Med. 2019;27:1–7. 31171019 10.1186/s13049-019-0636-0 PMC6555718

[19] Durrer B, Brugger H, Syme D. The medical on-site treatment of hypothermia ICAR-MEDCOM recommendation. High Alt Med Biol. 2003. 10.1089/152702903321489031. 12713717 10.1089/152702903321489031

[20] Strapazzon G, Procter E, Paal P, Brugger H. Pre-hospital core temperature measurement in accidental and therapeutic hypothermia. High Alt Med Biol. 2014. 10.1089/ham.2014.1008. 24950388 10.1089/ham.2014.1008

[21] Dow J, et al. Wilderness medical society clinical practice guidelines for the out-of-hospital evaluation and treatment of accidental hypothermia: 2019 update. Wilderness Environ Med. 2019;30:S47–69. 31740369 10.1016/j.wem.2019.10.002

[22] Shin J, Kim J, Song K, Kwak Y. Core temperature measurement in therapeutic hypothermia according to different phases: comparison of bladder, rectal, and tympanic versus pulmonary artery methods. Resuscitation. 2013. 10.1016/j.resuscitation.2012.12.023. 23306812 10.1016/j.resuscitation.2012.12.023

[23] Zafren K. Out-of-hospital evaluation and treatment of accidental hypothermia. Emerg Med Clin North Am. 2017;35:261–79. 28411927 10.1016/j.emc.2017.01.003

[24] Strapazzon G, et al. Influence of low ambient temperature on epitympanic temperature measurement: a prospective randomized clinical study. Scand J Trauma Resusc Emerg Med. 2015. 10.1186/s13049-015-0172-5. 26542476 10.1186/s13049-015-0172-5 PMC4635596

[25] Skaiaa SC, Brattebø G, Aßmus J, Thomassen Ø. The impact of environmental factors in pre-hospital thermistor-based tympanic temperature measurement: a pilot field study. Scand J Trauma Resusc Emerg Med. 2015. 10.1186/s13049-015-0148-5. 26400226 10.1186/s13049-015-0148-5 PMC4581419

[26] Doyle F, Zehner WJ, Terndrup TE. The effect of ambient temperature extremes on tympanic and oral temperatures. Am J Emerg Med. 1992. 10.1016/0735-6757(92)90003-G. 1616513 10.1016/0735-6757(92)90003-g

[27] Brown DJA, Brugger H, Boyd J, Paal P. Accidental hypothermia. N Engl J Med. 2012. 10.1056/NEJMra1114208. 23150960 10.1056/NEJMra1114208

[28] Raheja R, Puri VK, Schaeffer RC. Shock due to profound hypothermia and alcohol ingestion: report of two cases. Crit Care Med. 1981. 10.1097/00003246-198109000-00006. 7273811 10.1097/00003246-198109000-00006

[29] Baumgartner FJ, et al. Cardiopulmonary bypass for resuscitation of patients with accidental hypothermia and cardiac arrest. Can J Surg. 1992;35:184–7. 1562930

[30] Feiss P, Mora C, Devalois B, Gobeaux R, Christides C. Accidental deep hypothermia and circulatory arrest. treatment with extracorporeal circulation. Ann Fr Anesth Reanim. 1987. 10.1016/s0750-7658(87)80085-0. 3619158 10.1016/s0750-7658(87)80085-0

[31] Pasquier M, et al. Deep accidental hypothermia with core temperature below 24 °C presenting with vital signs. High Alt Med Biol. 2014. 10.1089/ham.2013.1085. 24527793 10.1089/ham.2013.1085

[32] Zafren K, et al. Wilderness medical society practice guidelines for the out-of-hospital evaluation and treatment of accidental hypothermia: 2014 update and International Commission for Mountain Emergency Medicine (ICAR MEDCOM) (Dr Zafren); the Faculty of Kinesiology and R. Wilderness Environ Med. 2014;25:S66–85. 25498264 10.1016/j.wem.2014.10.010

[33] Musi ME, et al. Clinical staging of accidental hypothermia: the revised swiss system: recommendation of the International Commission for Mountain Emergency Medicine (ICAR MedCom). Resuscitation. 2021;162:182–7. 33675869 10.1016/j.resuscitation.2021.02.038

[34] Frei C, et al. Clinical characteristics and outcomes of witnessed hypothermic cardiac arrest: a systematic review on rescue collapse. Resuscitation. 2019;137:41–8. 30771451 10.1016/j.resuscitation.2019.02.001

[35] Pasquier M, et al. Vitals signs in accidental hypothermia. High Alt Med Biol. 2020. 10.1089/ham.2020.0179. 33629884 10.1089/ham.2020.0179

[36] Anne Kelly C, Upex A, Bateman DN. Comparison of consciousness level assessment in the poisoned patient using the alert/verbal/painful/unresponsive scale and the Glasgow Coma Scale. Ann Emerg Med. 2004;44:108–13. 15278081 10.1016/j.annemergmed.2004.03.028

[37] McNarry AF, Goldhill DR. Simple bedside assessment of level of consiousness: comparison of two simple assessment scales with the Glasgow Coma Scale. Anaesthesia. 2004;59:34–7. 14687096 10.1111/j.1365-2044.2004.03526.x

[38] Mattu A, Brady WJ, Perron AD. Electrocardiographic manifestations of hypothermia. Am J Emerg Med. 2002;20:314–26. 12098179 10.1053/ajem.2002.32633

[39] Gill M, Martens K, Lynch EL, Salih A, Green SM. Interrater reliability of 3 simplified neurologic scales applied to adults presenting to the emergency department with altered levels of consciousness. Ann Emerg Med. 2007;49:403–8. 17141146 10.1016/j.annemergmed.2006.03.031

